# Mathematical Modeling of Subthreshold Resonant Properties in Pyloric Dilator Neurons

**DOI:** 10.1155/2015/135787

**Published:** 2015-04-16

**Authors:** Babak Vazifehkhah Ghaffari, Mojgan Kouhnavard, Takeshi Aihara, Tatsuo Kitajima

**Affiliations:** ^1^Malaysia-Japan International Institute of Technology, Universiti Teknologi Malaysia Kuala Lumpur, Jalan Semarak, 54100 Kuala Lumpur, Malaysia; ^2^Graduate School of Brain Sciences, Tamagawa University, 6-1-1 Tamagawagakuen, Machida, Tokyo 194-8610, Japan

## Abstract

Various types of neurons exhibit subthreshold resonance oscillation (preferred frequency response) to fluctuating sinusoidal input currents. This phenomenon is well known to influence the synaptic plasticity and frequency of neural network oscillation. This study evaluates the resonant properties of pacemaker pyloric dilator (PD) neurons in the central pattern generator network through mathematical modeling. From the pharmacological point of view, calcium currents cannot be blocked in PD neurons without removing the calcium-dependent potassium current. Thus, the effects of calcium (*I*_Ca_) and calcium-dependent potassium (*I*_KCa_) currents on resonant properties remain unclear. By taking advantage of Hodgkin-Huxley-type model of neuron and its equivalent RLC circuit, we examine the effects of changing resting membrane potential and those ionic currents on the resonance. Results show that changing the resting membrane potential influences the amplitude and frequency of resonance so that the strength of resonance (*Q*-value) increases by both depolarization and hyperpolarization of the resting membrane potential. Moreover, hyperpolarization-activated inward current (*I*_*h*_) and *I*_Ca_ (in association with *I*_KCa_) are dominant factors on resonant properties at hyperpolarized and depolarized potentials, respectively. Through mathematical analysis, results indicate that *I*_*h*_ and *I*_KCa_ affect the resonant properties of PD neurons. However, *I*_Ca_ only has an amplifying effect on the resonance amplitude of these neurons.

## 1. Introduction

When a low-amplitude sinusoidal input current having slowly changing frequency is given to neurons in the brain, the membrane potential peaks at a specific frequency in a subthreshold level. This oscillation phenomenon known as subthreshold resonance oscillation was first observed in myelinated nerves by Hermann [1]. Since then, it has been reported in many excitatory and inhibitory neurons in various regions of the central nervous system, such as squid giant axon [2, 3], inferior olive [4], thalamus [5], neocortex [6], entorhinal cortex [7], and hippocampal CA1 area [8, 9]. This resonance in neurons indicates that such neurons demonstrate frequency selectivity, which is a band-pass property. This property plays an essential role in the behavioral and perceptual functions in animals, but the effect of biophysical parameters on this phenomenon is poorly understood [10]. The importance of these resonant properties in neurons can be described from two points of views. One is from the relation between subthreshold resonance oscillation and synaptic plasticity [8], which is a fundamental mechanism of learning and memory in the brain [11]. The other is from the dependency of network oscillation frequency on the intrinsic-preferred frequencies of constituent neurons [12–14], which originated from the resonant properties of each neuron. These data suggest that understanding the mechanisms underlying the resonant properties of individual neurons is important to comprehend the behavior of neural networks and realize the scheme of brain signal processing.

Electrical circuit theory suggests that resonance arises from a combination of low- (RC) and high-pass filter (RL) properties that are closely related to ionic channel properties [14]. A low-pass filter depends on passive membrane property, while a high-pass filter is generated by slow voltage-dependent ion channels, such as hyperpolarization-activated potassium channels [9, 15, 16] and slow noninactivating potassium channels [6], which are activated in a low frequency range and decrease the amplitude of voltage change. The voltage in a low-pass filter always lags the input current wave, and its phase monotonically decreases as a function of frequency. However, the input current in a high-pass filter always lags the voltage wave, and the phase value remains positive. The coexistence of both high- and low-pass properties causes band-pass filtering (RLC circuit), which defines resonance.

Considering the advantages of RLC circuits (i.e., highly applicable even though the ionic current involved in the resonant properties is unknown), we used them to investigate not only the properties of resonant behavior, but also the roles of ionic currents in this inherited characteristic of pyloric dilator (PD) neurons.

Tohidi and Nadim [12] studied the roles of membrane resonance of pacemaker neurons on network frequency. They showed that network frequency in the carb pyloric central pattern generator is related to the stable oscillation produced by a group of pacemaker neurons: anterior burster (AB) and PD. Moreover, the experimental investigation of resonant properties of AB neurons is complicated by their small size. Thus, the authors only focused on PD neurons and showed that two currents play dominant roles in the resonant properties of these neurons. These currents are calcium current (*I*_Ca_) and hyperpolarized-inward current (*I*_*h*_). However, the roles of *I*_Ca_ and *I*_KCa_ and the effects of ionic channel parameters on resonance have not been discussed yet.

The present study focuses on the resonant properties of PD neurons to analyze the roles of ionic channel parameters on resonance and identify the effects of both *I*_Ca_ and *I*_KCa_ through theoretical analysis. In particular, we investigate the roles of voltage- and ionic-dependent channels in the subthreshold resonant properties of PD neurons through the Hodgkin-Huxley-type (HH-type) formulism and its equivalent electrical RLC circuit.

## 2. Method

### 2.1. HH-Type Dynamics of PD Neurons

**Figure 1:**
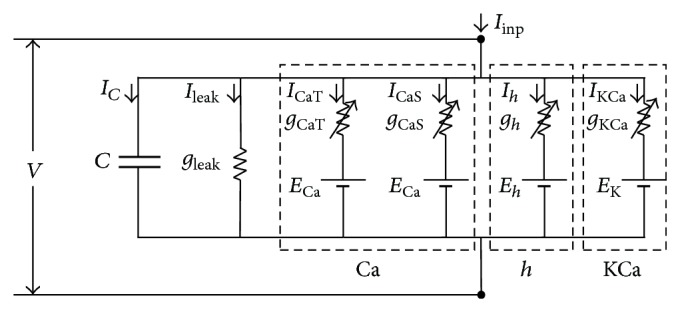
Conductance-based model of PD neuron. Dashed box denotes the individual ionic channels, that is, Ca (calcium channels (CaT and CaS)), *h* (hyperpolarization-activated channel), and KCa (calcium-dependent potassium channel).

Neurons usually contain multiple ionic channels with individual physiological properties. Neuronal behavior depends on the intrinsic properties of these ionic channels and their mutual interactions. Numerous complicated behaviors of neurons can be shown by HH formulism [16], which describes the dynamics of neuronal behavior in terms of ion currents through voltage-/ionic-gated channels and passive conductance. For PD neurons, the kinetic equations used to describe the activation and inactivation variables of ionic channels were initially introduced by Soto-Treviño et al. [17] and modified by Tohidi and Nadim [12] to adapt the HH-type model through the physiological behavior of these neurons, as shown in Figure 1.

The HH-type model includes a passive leak current (*I*_leak_), Ca^2+^-dependent K^+^ current (*I*_KCa_), hyperpolarization-activated inward current (*I*_*h*_), transient Ca^2+^ current (*I*_CaT_), persistent Ca^2+^ current (*I*_CaS_), and input current (*I*_inp_). The time evolution of the membrane potential (*V*), which followed the current conservation, is given by

(1) $$\begin{array}{c} C\frac{dV}{dt}=-I_{\text{leak}}-I_{h}-I_{\text{CaT}}-I_{\text{CaS}}-I_{\text{KCa}}+I_{\text{inp}}, \end{array}$$

where *V* is the membrane potential (mV), *t* is time (msec), *C* is the membrane capacitance (nF/cm^2^), and *I*_inp_ is a time-dependent input current. Each ionic current *I*_*i*_ depends on ion flow through channels whose permeability is controlled by activation (*m*) and/or inactivation (*h*) gating variables:

(2) $$\begin{array}{c} I_{i}=g_{i}m_{i}^{r}h_{i}^{q}\left(\;V-E_{i}\;\right),\;\left(i=h,\text{CaT},\text{CaS},\text{KCa},\text{leak}\right), \end{array}$$

where *g*_*i*_ is the maximum conductance, (*V* − *E*_*i*_) is the driving force, and  *r* and *q*  are integer values between 0 and 4 (Table 1). *E*_*i*_ is the Nernst potential for the particular ion. *I*_leak_ is obtained from *g*_leak_(*V* − *E*_*L*_) and the dynamics of the ion channel gating variables are given as follows:

(3) dmdtm∞V−mτmV,dhdt=h∞V−hτhV,

where *m*_*∞*_ and *h*_*∞*_ are the steady-state variables and *τ*_*m*_ and *τ*_*h*_ are the activation and inactivation time constants, respectively. In this model, *I*_KCa_ is rather complex, and its steady-state activation variable depends on both voltage and intracellular calcium concentration. The dependency on voltage and intracellular calcium concentration of each ionic current is shown in Table 1. In *I*_KCa_, the dependency of steady-state activation variable on intracellular calcium concentration [Ca^2+^] is described by

(4) $$\begin{array}{c} \frac{d\left[\text{C}{\text{a}}^{2+}\right]}{dt}=\frac{-F\left(I_{\text{CaT}}+I_{\text{CaS}}\right)-\left[\text{C}{\text{a}}^{2+}\right]+C_{0}}{\tau_{\text{Ca}}}, \end{array}$$

where *C*_0_ is the background intracellular calcium concentration, *τ*_Ca_ is the calcium buffering time constant, and *F* is a factor that translates the total calcium current into an intracellular concentration. A previous study [12] assumed that the reversal potential for calcium current *E*_Ca_ is dependent on intracellular calcium concentration. For the sake of simplicity in the present study, *E*_Ca_ is assumed to be a constant. Results show that this assumption does not affect the resonant properties of this neuron.

### 2.2. Equivalent Electrical RLC Circuit

As shown in Section 2.1, the dynamics of the neuron model are highly nonlinear functions of the membrane potential *V*. The resonant property of neurons depends on the linear properties of these dynamics; thus, the corresponding linear components need to be extracted from nonlinear dynamics. Various methods can be used for this extraction. Perturbation method is a well-known and highly efficient method for the linearization of nonlinear systems. For PD neurons, the membrane current (*I*) in its entirety is a sum of the leak current (*I*_leak_), capacitance current (*I*_*C*_), and active currents (*I*_Ca_, *I*_*h*_, and *I*_KCa_). In this section, the equivalent RLC circuit of each channel is obtained by linearizing their nonlinear dynamics. On the basis of the dependency of (in)activation variables on voltage and/or ionic current, ionic currents in PD neurons have two types. In the first group (CaT-, CaS-, and *h*-channels), the inactivation and activation variables only depend on the membrane potential. In the second group (with only one member, KCa-channel), the activation variable depends on both the membrane potential and intracellular calcium concentration, whereas the inactivation variable only depends on membrane potential.

#### 2.2.1. Impedance of CaT-, CaS-, and *h*-Channels

The same structure of linearization method is used for all channels in this group. Thus, we first describe the linearization method of transient calcium channel (CaT) and then only show the results of the linearized model for other currents. In the first step, we assume that the small variation of *I*_CaT_(*t*) from its equilibrium value (*I*_CaT_^∗^) is evaluated at *V* ^∗^, equilibrium potential, *δI*_CaT_(*t*):

(5) $$\begin{array}{c} I_{\text{CaT}}\left(\;t\;\right)={I_{\text{CaT}}}^{*}+\delta I_{\text{CaT}}\left(\;t\;\right). \end{array}$$

Let the small variations in activation variable [*m*_CaT_(*t*)], steady-state [*m*_CaT_^∗^(*t*)], and time constant  [*τ*_*m*_CaT__(*t*)]  be *δm*_CaT_(*t*), *δm*_CaT_^∗^, and *δτ*_*m*_CaT__, respectively. Moreover, let the small variations in the inactivation variable [*h*_CaT_(*t*)], steady-state [*h*_CaT_^∗^(*t*)], and time constant  [*τ*_*h*_CaT__(*t*)]  be *δh*_CaT_(*t*), *δh*_CaT_^∗^, and *δτ*_*h*_CaT__, respectively. The following relation is obtained from (2) for the CaT-channel and (5):

(6) ICaT∗+δICaT=g−CaT·mCaT∗+δmCaT3·hCaT∗+δhCaT·V∗+δV−ECa,

where *X* ^∗^ stands for the quantity *X* evaluated at *V* ^∗^. Thus, *I*_CaT_ in equilibrium state can be expressed as

(7) $$\begin{array}{c} {I_{\text{CaT}}}^{*}={\overset{-}{g}}_{\text{CaT}}\cdot {m_{\text{CaT}}^{*}}^{3}\cdot h_{\text{CaT}}^{*}\cdot \left(\;V^{*}-E_{\text{Ca}}\;\right). \end{array}$$

Substituting (7) into (6) and neglecting high-order terms over second-order terms, we can obtain *δI*_CaT_ by

(8) δICaT=g¯CaT·mCaT∗3·δhCaT·V∗−ECa+g¯CaT·3δmCaT·mCaT∗2·hCaT∗·V∗−ECa+g¯CaT·mCaT∗2·hCaT∗·δV.

Based on (3), the following relation can be obtained by assuming the small variation:

(9) dxCaT∗+δxCaTdt=xCaT∞∗+δxCaT∞−xCaT∗+δxCaTτx,CaT∗+δτx,CaTWWWeWiWWWWWWx=m,h=xCaT∞∗−xCaT∗+δxCaT∞−δxCaT ·1τx,CaT∗·1−1τx,CaT∗·δτx,CaT+⋯.

Neglecting high-order terms over second-order terms, we can write (9) as

(10) dδxCaTdt1τx,CaT∗·δxCaT∞−δxCaT=1τx,CaT∗·dxCaT∞VdV∗·δV−δxCaT,

where *dX*/*dV*|_∗_ shows the quantity of (*dX*/*dV*) estimated at the equilibrium potential *V* ^∗^. For convenience purposes, the notation of the derivative *d*/*dt* is replaced by the operator symbol  *p*.  The following relations are obtained by replacing *x* with *m* and *h*:

(11) δmCaT1/τm,CaT∗·dmCaT∞/dV∗p+1/τm,CaT∗·δV,

(12) δhCaT=1/τh,CaT∗·dhCaT∞/dV∗p+1/τh,CaT∗·δV.

By substituting (11) and (12) into (8), we can express the variation of *I*_CaT_ as

(13) δICaT=g¯CaT·mCaT∗3·1/τh,CaT∗·dhCaT∞/dV∗p+1/τh,CaT∗WW·V∗−ECa+g¯CaT·3WW·1/τm,CaT∗·dmCaT∞/dV∗p+1/τm,CaT∗WW·1/τh,CaT∗·dhCaT∞/dV∗p+1/τh,CaT∗mCaT∗2·hCaT∗·V∗−ECa+g¯CaT·mCaT∗3·hCaT∗W·δV.

That is,

(14) δICaTδV=g¯CaT·mCaT∗3·hCaT∗+g¯CaT·mCaT∗3·1/τh,CaT∗·dhCaT∞/dV∗p+1/τh,CaT∗·V∗−ECa+g¯CaT·3·mCaT∗2·hCaT∗·1/τm,CaT∗·dmCaT∞/dV∗p+1/τm,CaT∗·V∗−ECa.

**Figure 2:**
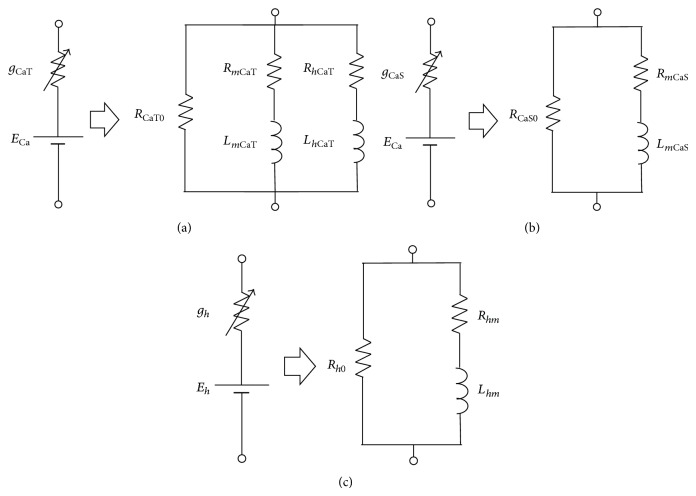
Equivalent model of first group of ionic channels. The model of (a) CaT-, (b) CaS-, and (c) *h*-channels (all left traces). The equivalent RL circuit model of (a) CaT-, (b) CaS-, and (c) *h*-channels (all right traces) for small input perturbation. The details of each component in the equivalent circuit models are shown in Appendix A.1, A.2, and A.3.

Equation (14) indicates the admittance of the CaT-channel, which should be reversed to obtain the impedance. Thus, the first term of (14) can be considered as the inverse of the resistor and the second and third terms as the inverse of the series impedance of the resistor and the inductor. For small input perturbation, the model of the CaT-channel can be figured by the equivalent RL circuit model, as shown in Figure 2(a) (right trace). Similarly, the admittance of persistent calcium (CaS) and hyperpolarization-activated (*h*) channels can be obtained as follows, respectively:

(15) δICaSδV=g¯CaS·mCaS∗3+g¯CaS·3·mCaS∗2·1/τm,CaS∗·dmCaS∞/dV∗p+1/τm,CaS∗·V∗−ECa,

(16) δIhδV=g¯h·mh∗+g¯h·1/τm,h∗·dmh∞/dV∗p+1/τm,h∗·V∗−Eh.

In Figures 2(b) and 2(c) (right traces), the models of CaS- and *h*-channels are, respectively, shown as an RL circuit.

#### 2.2.2. Impedance of Ca^2+^-Dependent K^+^ Channel

All the variables of the calcium (CaT and CaS) and hyperpolarization-activated potassium (*h*) channels depend only on the membrane potential or time. However, for the KCa-channel, the activation variable also depends on intracellular calcium concentration. To obtain the equivalent RL circuit of this channel, let the small variation of *I*_KCa_(*t*) from its equilibrium value (*I*_KCa_^∗^) be *δI*_KCa_(*t*):

(17) $$\begin{array}{c} I_{\text{KCa}}\left(\;t\;\right)={I_{\text{KCa}}}^{*}+\delta I_{\text{KCa}}\left(\;t\;\right). \end{array}$$

In addition, let the small variations of *m*_KCa_ and Ca be *δm*_KCa_ and *δ*Ca, respectively. Afterward, applying the same process as described in Section 2.2.1 to (2) and (3) for the KCa-channel and (4) results in the following relations:

(18) δIKCa=g¯KCa·4·mKCa∗3·V∗−EK·δmKCa+g¯KCa·mKCa∗4·δV,

(19) δmKCa=1p+1/τKCa∗·1τKCa∗·dmKCa∞dV∗·δV+dmCaS∞dV∗·δCa,

(20) δCa=−FτKCa·p+1/τKCa·δIKCa.

Substituting (19) and (20) into (18), we can express the admittance of the KCa-channel as

(21) δIKCaδV=g¯KCa·mKCa∗4+g¯KCa·4·mKCa∗3·1τm,KCa∗W·V∗−EK·dmKCa∞dV∗·p+1τm,KCa∗−1W−g¯KCa·g¯CaS·4·mCaS∗3·mKCa∗3·1τm,KCa∗WWW·1τCa·V∗−EK·dmKCa∞dV∗·F·−g¯KCaWWW·g¯CaT·4·mCaT∗3·mKCa∗3·hCaT∗·1τm,KCa∗WWW·1τm,KCa∗1τCa·V∗−EK·dmKCa∞dV∗·FW·p+1τm,KCa∗−1·p+1τCa−1−g¯KCa·g¯CaSW·12·mCaS∗2·mKCa∗3·1τm,KCa∗·1τCa·1τm,CaSW·V∗−ECa·dmKCa∞dV∗·dmCaS∞dV∗W·F×p+1τm,KCa∗−1·p+1τCa−1W·p+1τm,CaS−1−g¯KCa·g¯CaT·4·mCaT∗3W·mKCa∗3·1τm,KCa∗·1τCa·1τh,CaTW·V∗−ECa·V∗−EK·dmKCa∞dV∗·dhCaT∞dV∗W·F×p+1τm,KCa∗−1·p+1τCa−1W·p+1τh,CaT−g¯KCa·g¯CaT·12·mCaT∗2·hCaT∗W·mKCa∗3·1τm,KCa∗·1τCa·1τm,CaTW·V∗−ECa·V∗−EK·dmKCa∞dV∗·dmCaT∞dV∗W·F×p+1τm,KCa∗−1·p+1τCa−1W·p+1τm,CaT.

**Figure 3:**
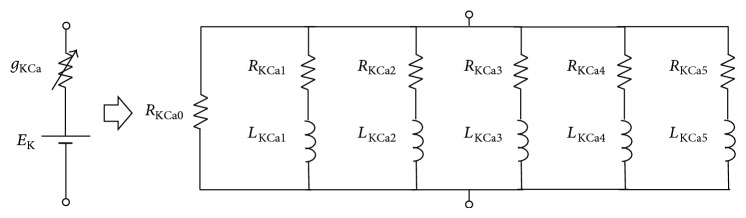
Equivalent model of first group of ionic channels. The model of (a) CaT-, (b) CaS-, and (c) *h*-channels (all left traces). The equivalent RL circuit model of (a) CaT-, (b) CaS-, and (c) *h*-channels (all right traces) for small input perturbation. The details of each component in the equivalent circuit models are shown in Appendix A.1, A.2, and A.3.

**Figure 4:**
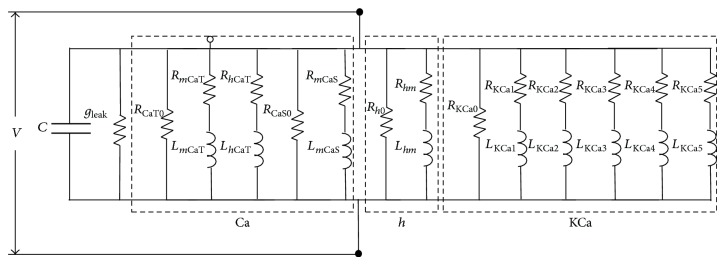
Equivalent electrical RLC circuit for a compartmental neuron model with CaT-, CaS-, *h*-, and KCa-channels.

Equation (21) represents the admittance of the KCa-channel. The impedance can be obtained by reversing this admittance. Thus, the first term of (21) can be considered as the inverse of the resistor and the other terms as the inverse of the series impedance of the resistor and the inductor. The model of the KCa-channel can be figured by the equivalent RL circuit model as shown in Figure 3 (right trace). Therefore, the equivalent electrical RLC circuit of a compartmental neuron model that includes the CaS-, CaT-, *h*-, and KCa-channels can be obtained by combining all the equivalent RL circuits for individual channels and passive membrane RC circuit as shown in Figure 4.

The membrane potential of the equivalent RLC circuit *v* (where *v* = *V* ^∗^ − *V*) can be obtained by solving the following simultaneous equation:

(22) Cdvdt=−gLeak−1RCaT0−1RCaS0−1Rh0−1RKCa0·vCdvdt=−ImCaT−IhCaT−ImCaS−Imh−IKCa1−IKCa2Cdvdt=−IKCa3−IKCa4−IKCa5+Iinp,Ly·dIydt=v−Ry·Iy,y=mCaT,hCaT,mCaS,hm,KCa1,KCa2,KCa3,EKCa4,KCa5,

where *L*_*y*_ and *R*_*y*_ are the inductor and the resistor of each ion channel in the equivalent RLC circuit and *I*_*y*_ is a current that fluxes through ion channels.

## 3. Simulation

Impedance analysis is conventionally employed to investigate the subthreshold resonant properties of a compartmental neuron model. In impedance analysis, the sinusoidal current with linearly changing frequency,* Chirp* [18] or* ZAP* [19] current, is injected into the neuron and the frequency preference of neurons is examined. In the present study, we input the* Chirp* current into the HH-type model and its equivalent RLC circuit to analyze the voltage response and impedance profile of PD neurons. The* Chirp* input current is described as

(23) $$\begin{array}{c} I_{\text{inp}}=I_{\text{amp}}\sin\left(\;2\pi f\left(\;t\;\right)t\;\right), \end{array}$$

where the time-dependent frequency *f*(*t*) increases from *f*_0_ = 0 Hz to *f*_max⁡_ = 5 Hz for a total duration *T* of 10 s, which is expressed by

(24) $$\begin{array}{c} f\left(\;t\;\right)=f_{0}+\left(\;f_{\max}-f_{0}\;\right)\left(\;\frac{t}{2T}\;\right). \end{array}$$

As the resonance of PD neuron occurs in lower frequency (~<2 Hz), in our analysis, very small steps are used for sampling to make a comprehensive sweeping frequency in given range. The impedance profile can be obtained as a ratio of the fast Fourier transforms (FFTs) of voltage response and input ZAP current, given by

(25) $$\begin{array}{c} Z\left(\;f\;\right)=\frac{\text{FFT}\left(V\right)}{\text{FFT}\left(I\right)}, \end{array}$$

where FFT(*V*) and FFT(*I*) are the fast Fourier transforms of the voltage response and the input current, respectively. Impedance is a complex quantity, where the real part (*Z*_*Re*_) is the resistance and the imaginary part (*Z*_*Im*_) is the reactance. It can also be shown as a vector that includes magnitude (|*Z*(*f*)|) and phase (*φ*(*f*)) as a function of frequency, which can be expressed as

(26) ZfZRe2+ZIm2,

(27) φf=tan−1ZImZRe.

In this study, frequencies below 0.1 Hz are not shown in the magnitude and phase profiles to avoid irregular distortions of results at a low frequency. Model simulation is performed through the MATLAB implementation of the numerical solution method based on the Runge-Kutta fourth-order method. Results show that the impedance magnitude (IM) of the HH-type model is associated with noise caused by software restrictions and errors. These errors are removed by using the local regression method [20]. Although the Nernst potential of calcium channels (*E*_Ca_) is a dependent variable of intracellular calcium concentration in physiological experiments [12], it is considered as a constant value. Our results show that this alternation does not affect the resonant properties of the HH-type model of PD neurons. The parameters and constants used in the following simulations are listed in Table 2. We call the situation control condition when these values are used in simulations.

## 4. Result and Discussion

Various types of ionic channels involved in the generation of subthreshold resonance oscillation in PD neurons [12]. However, the effects of these ionic channels, particularly calcium channels and calcium-dependent potassium channels, on the resonant properties of PD neurons are poorly understood. From a physiological point of view, calcium currents cannot be blocked without also removing calcium-dependent potassium currents. Thus, we examine the effects of these unexplored ionic channels together with *h*-channel on the resonant properties of PD neurons on the basis of dynamical system theory.

**Figure 5:**
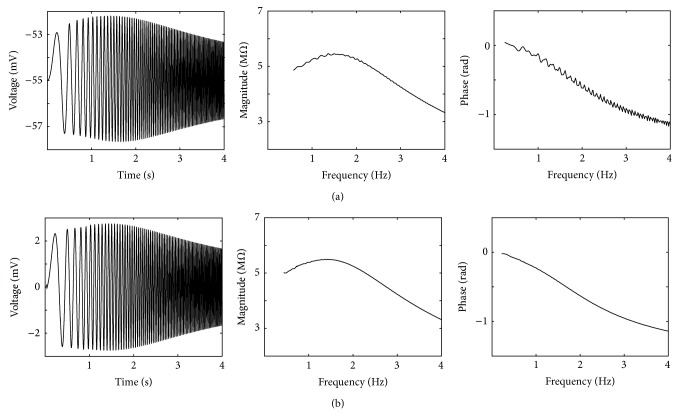
Comparison between the HH-type model and the equivalent RLC circuit model. (a) The voltage response and impedance profile of HH-type model under control condition. (b) The voltage response and impedance profile of equivalent RLC circuit model.

The voltage response and impedance profile by giving the Chirp current input to both the HH-type model and the equivalent RLC circuit model are shown in Figure 5. The result shows that the main features of membrane resonance of both models such as resonance frequency, behavior of phase profile, amplitude of voltage profile, and the resonance magnitude in the impedance profile are almost the same. It means the equivalent RLC circuit accurately describes the subthreshold resonance behavior of the PD neuron model.

Moreover, to investigate the resonant properties of both HH-type model and its equivalent RLC circuit, we divide this section into two parts: (1) voltage dependency and ionic mechanisms in the HH-type model and (2) roles of ionic currents in equivalent RLC circuit.

In the first part, we examine the dependency of subthreshold resonance on the membrane resting potential and ionic currents using the HH-type model of PD neurons. This evaluation is based on the detailed comparison of voltage response and impedance profile for each condition during the* Chirp* current input. In the second part, we examine the effects of ionic channels on subthreshold resonant properties using the equivalent electrical RLC circuit of the HH-type model. In particular, the ionic currents are removed initially to compare the roles of these currents in both the HH-type model and the equivalent RLC circuit. Then, we alter the maximum conductance of each ionic channel and investigate the effects of these alternations on the voltage response and impedance profile of the equivalent RLC circuit.

### 4.1. Voltage Dependency and Ionic Mechanisms in HH-Type Model

**Figure 6:**
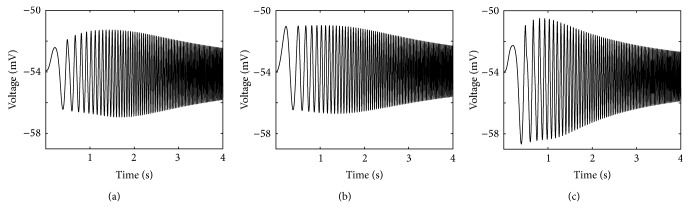
The roles of *I*_*h*_ and *I*_Ca_ in membrane resonance of PD neurons. (a) Control condition. (b) Blocking *I*_Ca_ mainly affected the upper envelope of the voltage profile. (c) Blocking *I*_*h*_ dramatically changed the lower envelope of voltage profile. The local minimum value disappeared after blocking *I*_*h*_.

The HH-type model of PD neurons, including *h*-, CaT-, CaS- and KCa-channels, is considered in this study. Tohidi and Nadim [12] investigated the ionic roles in the membrane resonant property of PD neurons by using CS^+^ and MN^2+^ blockers to block *I*_*h*_ and *I*_Ca_, respectively. They showed that the membrane resonance of PD neurons is strictly directed by *I*_*h*_ and *I*_Ca_, such that blocking *I*_*h*_ changes the lower envelope of the voltage profile by removing its local minimum value. By contrast, blocking *I*_Ca_ changes the upper envelope of the voltage profile. We simulate their results by using the HH-type model of PD neurons (Figure 6).

Using linearization methods, we show that *I*_Ca_ has an amplifying role in the resonant properties of PD neurons. However, the combination of *I*_Ca_ and *I*_KCa_ has an effect on resonant properties. This behavior occurs because *I*_KCa_ possesses negative feedback properties, which will be discussed in the next section. Here, first, we show the effects of changing resting potential on the subthreshold resonant properties of PD neurons under control conditions.

Then, the role of ionic mechanisms in both hyperpolarized and depolarized membrane potentials is investigated by replacing each maximum conductance of these currents with zero in different resting potentials.

**Figure 7:**
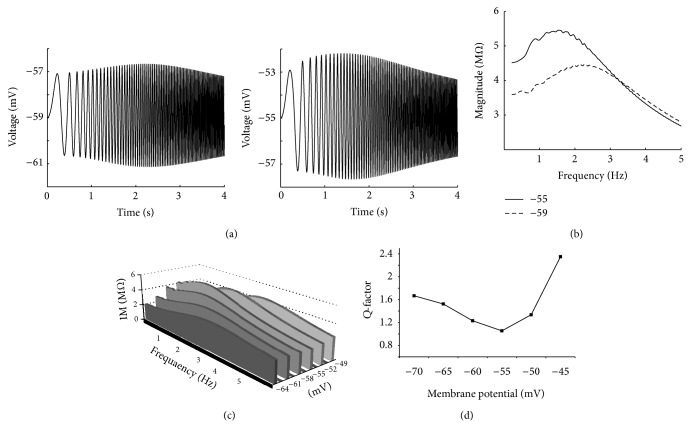
The response of HH-type model to the Chirp input. (a) The voltage response at the resting membrane potential of −59 mV (left trace) and −55 mV (right trace). The peak-to-peak amplitude of voltage response is reduced and shifted to right by decreasing the resting membrane potential. (b) Impedance magnitude. Both the maximum impedance magnitude (|*Z*|_max⁡_) and resonance frequency (*f*_res_) depend on the resting membrane potential. (c) Three-dimensional plot showing the impedance magnitude plot (*Z*-*f* curve) at different resting potentials. (d) The values of *Q*-factor at different resting potentials.

Figures 7(a)–7(d) show the effects of changing resting potential on the subthreshold resonant properties. The value of resting potential under control condition (−55 mV) is changed, and both voltage response and impedance profiles are compared. At −55 mV (Figure 7(a), right trace), the maximum amplitude of the voltage profile emerges at low frequencies, which agrees with experimental results [12].

IM (|*Z*|_max⁡_) and resonance frequency (*f*_res_) are also changed for different resting potentials (Figure 7(b)).  Figure 7(c) shows the 3D plot of the IM curves obtained at various resting potentials (between −64 and −49 mV). A comparison of the IM curves shows that the maximum IM emerges at approximately −55 mV. In addition, the IMs are reduced in both depolarizing and hyperpolarizing directions. We also examine the effect of changing resting potential on the strength of resonance using the *Q*-factor, which is expressed by the ratio between maximal impedance (|*Z*(*f*_res_)|) and impedance at the lowest frequency (|*Z*(0.5)|). This ratio implies the sharpness of the impedance curve around the resonance frequency. Based on our results, the quantitative criterion of the *Q*-factor is assigned to *Q* ≥ 1.01, which indicates that the maximal impedance should be at least 1% higher than the minimal impedance (|*Z*(0.5)|) to show the resonant properties of the model. As shown in Figure 7(d), the resonant behavior depends on two membrane potential ranges. This behavior is prominent by depolarizing the membrane potential (e.g., the *Q*-factor is 2.35 near −50 mV) and decreases to the minimum value by approaching the resting potential (−55 mV). This behavior is almost the same as what was observed in CA1 neurons [19].

Conversely, the strength of resonance increases as the membrane potential is hyperpolarized from 1.055 to 1.67. This U-shaped voltage dependence is clear in the magnitude of impedance profile (e.g., for hyperpolarizing membrane potential, see Figure 7(b)). Therefore, two voltage ranges arise, where the resonance frequency is prominent and the *Q*-factor shows opposite voltage dependence.

**Figure 8:**
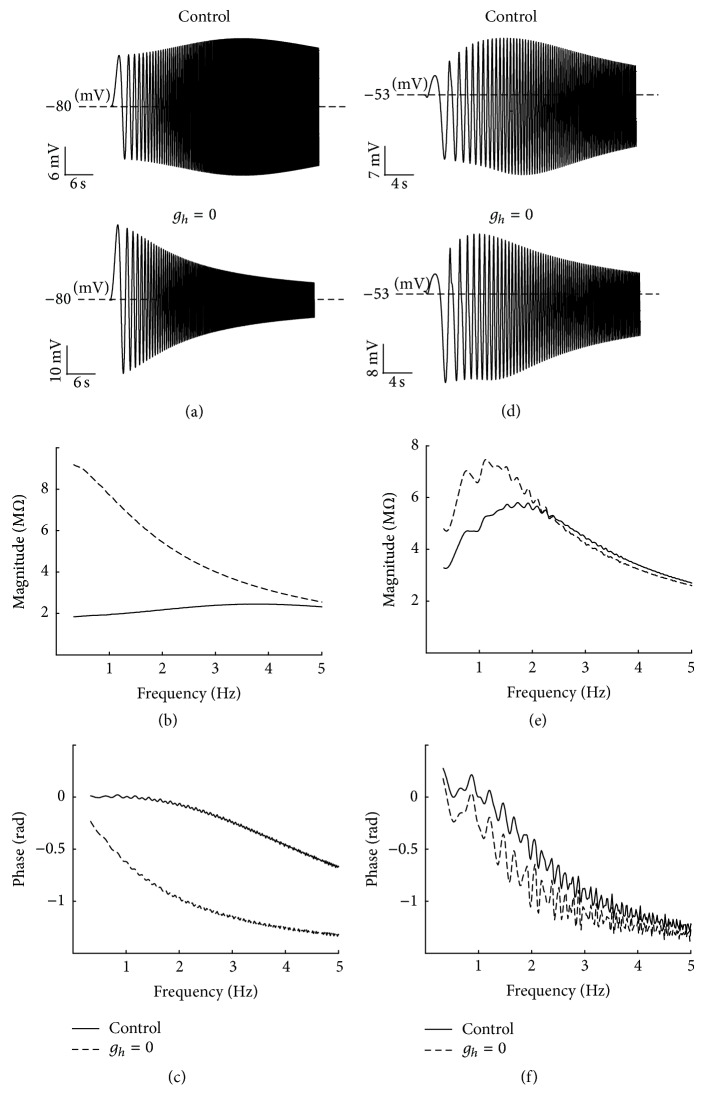
Effects of removing *g*_*h*_ on resonance at different resting potentials. (a) Voltage response to the Chirp input at the hyperpolarized resting potential (−80 mV) under control condition (upper trace) and after removing *g*_*h*_ (lower trace). (b) Impedance magnitude, as a function of frequency. At hyperpolarized resting potential, both the impedance magnitude (|*Z*|_max⁡_) and resonance frequency (*f*_res_) disappeared by removing *g*_*h*_. (c) In the phase profile, the resonant property is removed by removing *g*_*h*_ at hyperpolarized resting potential. (d) Voltage response at the depolarized resting potential (−53 mV) under control condition (upper trace) and after removing *g*_*h*_ (lower trace). (e) At depolarized resting potential, by removing *g*_*h*_, the impedance magnitude is increased and resonance frequency is transferred to the lower frequencies. (f) In the phase profile, the resonant property is decreased at depolarized resting potential. The fluctuating form of phase curve is caused by ionic channels properties (high nonlinearity of HH-type dynamics of PD neurons).

Based on the behavior of the inactivation and activation variables of ionic channels in PD neurons, two specific ionic channels present the biophysical resonant properties of PD neurons: *h*-channel and Ca^2+^- (CaS- and CaT-) channels (in association with KCa-channel). The contribution of these currents to PD neuron resonance is determined by using the HH-type model of this neuron.  Figure 8 shows the voltage responses and impedance profiles for the HH-type model without *h*-channel.  Figure 8(a) indicates that removing *h-*channel (*g*_*h*_ = 0) at hyperpolarized resting potentials removes the resonant properties completely and increases the impedance in the low frequency range (Figure 8(b)). The *Q*-factors at hyperpolarized resting potentials before and after removing *h*-channel are *Q* = 1.28 and 0, respectively. Phase of impedance is also significantly changed (Figure 8(c)), phase is reduced, and positive lags in the low frequency range are vanished. Moreover, the phase curve is transformed to a monophase function. These results indicate that *h*-channel also has a dominant role on resonant properties at hyperpolarized resting potentials. Interestingly, removing *h*-channel also affects both voltage response (Figure 8(d)) and magnitude of impedance (Figure 8(e)), such that it removes the resonant property in the lower trace of voltage response and increases the impedance magnitude.

This asymmetrical change in voltage response occurs for membrane resting potentials higher than −55 mV because *h*-channel is mostly activated in the hyperpolarized membrane potential range and has a smaller effect on resonant properties than Ca^2+^- (CaS- and CaT-) channels (in association with KCa-channel) in this range. However, *h*-channel is semiactivated in membrane potential lower than −53 mV at the depolarized level but inactivated in membrane potential higher than −53 mV. Thus, it has a modest effect on resonance magnitude and frequency, as shown in Figure 8(e). Removing *h*-channel also decreases the resonant property of phase profile in this range (Figure 8(f)). Considering all aforementioned results and the decrease in *Q*-value after removing *h*-channel (*Q* = 1.24 for control condition and 1.08 for removing *h*-channel), we suggest that *h*-channel affects the resonant properties of PD neurons at depolarized potentials.

This property of *h*-channel in PD neurons is in contrast to what has been reported for CA1 pyramidal neurons, for which the resonant properties at depolarized potentials do not depend on this current [21].

**Figure 9:**
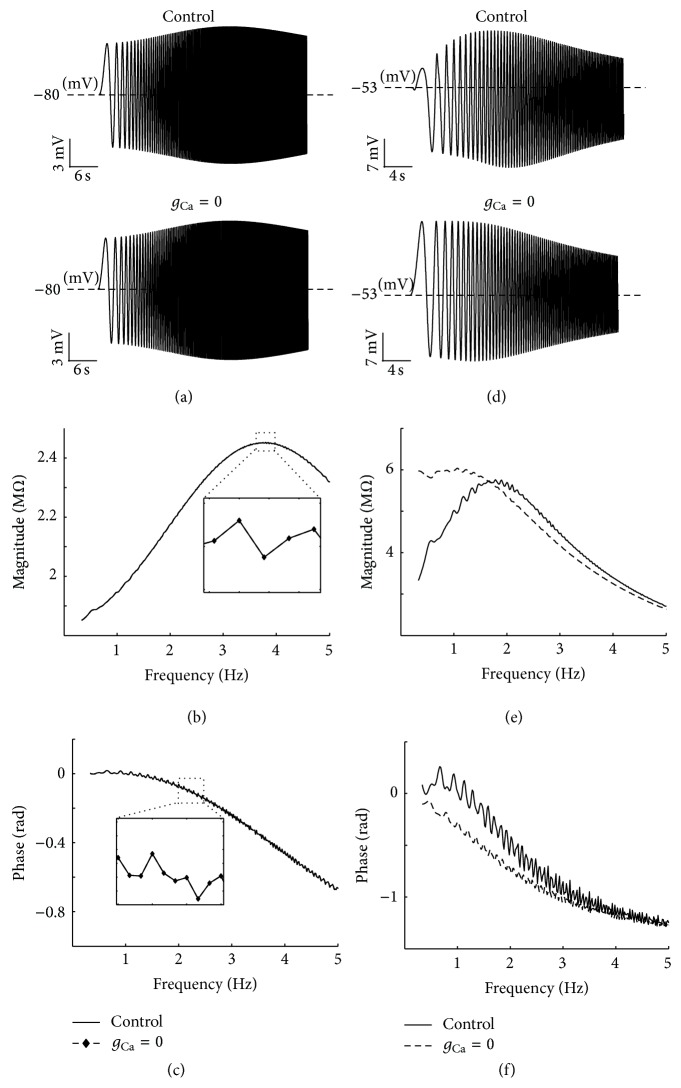
Effects of removing *g*_Ca_ on resonance at different holding potentials. Removing *g*_Ca_ has no effect on (a) voltage response, (b) impedance magnitude, or (c) phase profile at hyperpolarized membrane potentials. However, at depolarized membrane potentials removing *g*_*C*_, (d) abolish the resonance in upper trace of voltage response and also manipulate the lower trace. Removing *g*_Ca_ also mainly removes the resonance in (e) impedance. It means that these channels are dominant factor in resonance at depolarized voltages. (f) Removing *g*_Ca_ decreases the resonant properties of phase at depolarized voltages. The fluctuating form of phase curve (f) is caused by ionic channels properties (high nonlinearity of HH-type dynamics of PD neurons).

A possible role of Ca^2+^- (CaS- and CaT-) channels in resonant properties is evaluated by removing Ca^2+^-channels (set *g*_Ca_ = 0) from the HH-type model. As shown in Figure 9, the resonant properties at hyperpolarized membrane potentials are not manipulated by Ca^2+^-channels (Figures 9(a), 9(b), and 9(c)). This finding means that *h*-channel is a dominant factor that influences the resonant properties of PD neurons in this range of resting membrane potentials (Figures 8(a), 8(b), and 8(c)). Conversely, removing Ca^2+^-channels at depolarized membrane potentials completely abolishes the resonance in the upper trace and manipulates the lower trace of voltage response (Figure 9(d)). It also changes the resonance frequency (Figure 9(e)) and phase profile (Figure 9(f)), such that the resonance frequency is transferred to the low frequency ranges and the phase curve changes into a mono-phase-like function. Considering these results and the decrease in *Q*-values to a level lower than the threshold after removing Ca^2+^-channels (*Q* = 1.24 for control and 1.03 for removing Ca^2+^-channels), we suggest that Ca^2+^-channels (in association with KCa-channel) are a dominant factor that influences the resonant properties of PD neurons at the depolarized voltage range. However, removing Ca^2+^-channels also inactivates KCa-channel. Thus, determining which of these currents plays a resonator role in the PD neurons remains ambiguous. To resolve this ambiguity, the equivalent RLC circuit of PD neurons is considered for further investigation of the roles of these currents on resonance.

### 4.2. Roles of Ionic Currents in Equivalent RLC Circuit Model

In the subthreshold regime, the equivalent RLC circuit is an extremely useful tool to investigate the resonant properties for various types of neurons. In this regime, the effects of membrane nonlinearities are not significant [22]. Thus, the equivalent RLC circuit can be assumed as a broad theoretical method to examine not only the properties of resonant behavior, but also the roles of ionic current mechanisms in this inherited characteristic of many neurons.

No distinct relation exists between the biophysical properties of ionic currents and the components of equivalent RLC circuit. Thus, this powerful tool is marginalized. However, we suggest that the effect of individual ionic currents on resonant properties of PD neurons can be fully examined through the equivalent RLC circuit model by considering the inverse relationship between the maximum conductance of different voltage- and/or ionic-gated channels and the components (resistances and inductances) of equivalent RLC circuit model (the Appendix). Before considering this relationship, we show some results to adequately address the prevailing problem.

First, the differential contributions of ionic currents to resonance at various resting membrane potentials are investigated through the equivalent RLC circuit model. At hyperpolarized membrane potentials, Ca^2+^-channels removal has no effect on voltage response and impedance profile, whereas the elimination of *h*-channel abolishes resonance (results are not shown). These results are very close to the results of the HH-type model (Figures 8(a), 8(b), 8(c), 9(a), 9(b), and 9(c)). However, at depolarized membrane potentials (i.e., −53 mV), setting the maximum conductance of *h*-channel (*g*_*h*_ = 0) to zero abolishes resonance, and removing Ca^2+^-channels has no effect on resonance. In this study, we show that the equivalent RLC circuit model can be considered as a powerful tool to examine the resonant properties of neurons with Ca^2+^-channels and KCa-channel (the activation of one channel depends on the activation of another one), by reducing the value of inductive of resonator component (increasing *g*_KCa_, maximum conductance of KCa-channel). This change transforms the resonance frequency from lower to higher ranges for both equivalent RLC circuit and HH-type model. This transformation has two advantages. First, we visualize the resonance of hidden resonator element (i.e., in the case of reducing *g*_*h*_). Second, we can investigate the role of each voltage- and/or ionic-gated current through the branches of the equivalent RLC circuit model (Figure 4) without losing the resonance. Changing the maximum conductance of ionic channels also changes the quantities of resonance (*Q*-value, magnitude of impedance profile, resonance frequency, etc.). However, it fully discloses the effect of each ionic current on resonant properties (which is the main scope of this study).  Table 3 shows the values of all components of the equivalent RLC circuit before setting the desired maximum conductance to a large value, where some resistances and inductances take negative values. These series circuits of negative inductance and resistance can be replaced by parallel circuit with a positive resistance and a series circuit of positive resistance and capacitance. This replacement for negative resistance and inductance elements helps us to model the equivalent electrical RLC circuit of PD neurons using hardware components [23] and to uncover the capacitor-like behavior of these channels. This result indicates that they act as a low-pass filter unlike the resonator currents that play a high-pass filter role in the resonant properties of neurons [24]. Specifically, it means that positive feedback exists between calcium conductance and membrane depolarization. Thus, Ca^2+^-channels amplify the resonant properties of PD neurons, which contradict previous experimental results. Considering that Ca^2+^-channels exert no effect on resonance, we determine why the resonant properties of PD neurons are changed by removing Ca^2+^-channels in physiological experiments.

As shown in Table 3, at resting membrane potential (−55 mV) in control condition, the negative elements are *L*_KCa1_ = −5 (H) and *R*_KCa1_ = −83.27 (MΩ) for KCa-channel and *L*_*m*CaT_ = −0.0152 (H), *R*_*m*CaT_ = −2.98 (MΩ), *L*_*m*CaS_ = −0.0077 (H), and *R*_*m*CaS_ = −0.59 (MΩ) for Ca^2+^-channels (CaT- and CaS-channels). The negative resistors and inductances in Ca^2+^-channels only depend on the term of *V* − *E*_Ca_ ((A.3) and (A.4) in the Appendix). To understand the physiological aspect of this relation, it should be considered that the extracellular concentration of Ca^2+^ ions is higher than the intracellular concentration. This condition results in the quite large positive Nernst potential of calcium channels, *E*_Ca_. Therefore, *V* − *E*_Ca_ takes the large negative value and, subsequently, the impedance of the calcium channel becomes negative. For the KCa-channel, the negativity of *L*_KCa1_ and *R*_KCa1_ depends on the following terms: *V* − *E*_K_,  *V* − *E*_Ca_, (1/*τ*_Ca_ − 1/*τ*_*m*KCa_^∗^), (1/*τ*_*m*CaS_^∗^ − 1/*τ*_*m*KCa_^∗^), (1/*τ*_*m*CaT_^∗^ − 1/*τ*_*m*KCa_^∗^), and (1/*τ*_*h*CaT_^∗^ − 1/*τ*_*m*KCa_^∗^) ((A.5) to (A.7) in the Appendix). Our results show that the negativity of *L*_KCa1_ and *R*_KCa1_ is mostly determined by the negativity of *V* − *E*_Ca_ and (1/*τ*_Ca_ − 1/*τ*_*m*KCa_^∗^). From a physiological point of view, the negativity of (1/*τ*_Ca_ − 1/*τ*_*m*KCa_^∗^) is attributed to the fact that the time constant of the KCa-channel (*τ*_*m*KCa_^∗^) is smaller than that of calcium buffering (*τ*_Ca_, which is the sum of diffusion, buffering, and calcium pumps).

**Figure 10:**
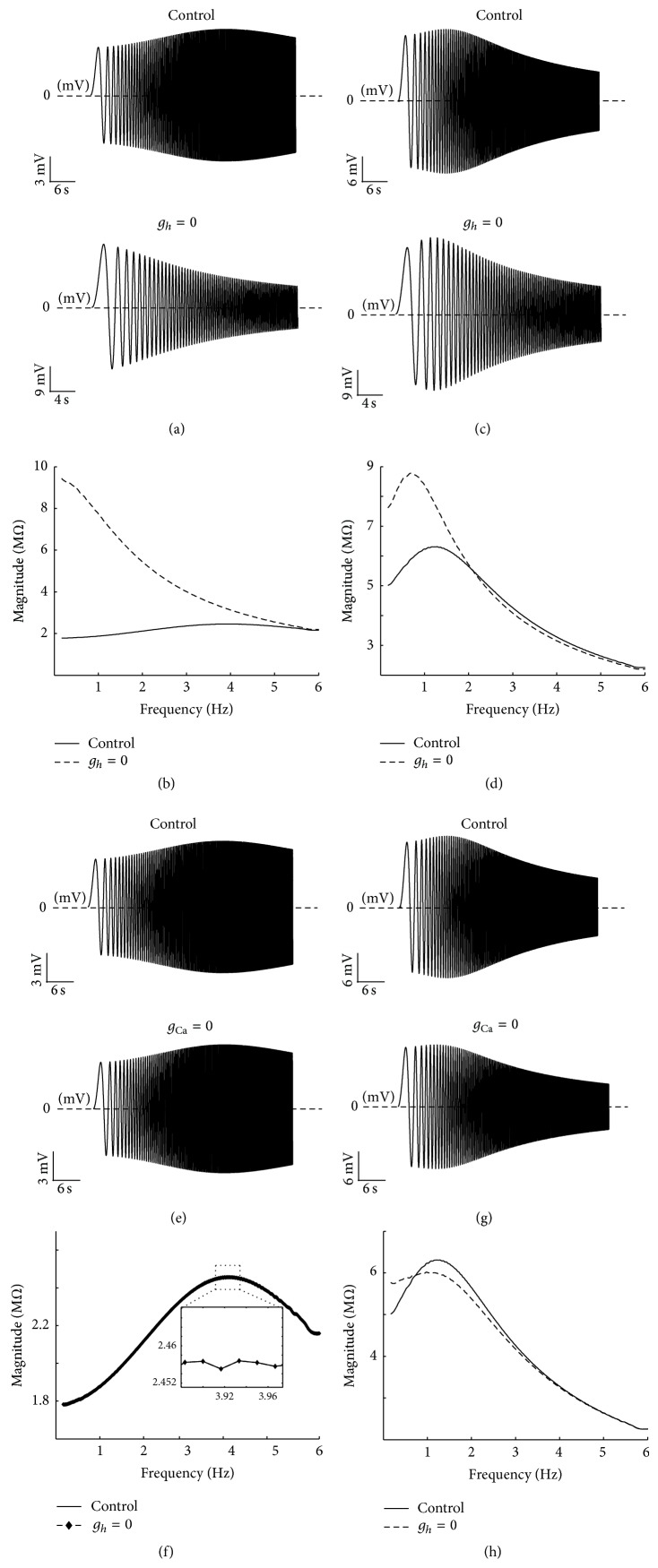
The effects of removing *h*- and Ca^2+^-channels on resonance at different resting membrane potentials for equivalent RLC circuit model. (a) The voltage responses at the hyperpolarized resting membrane potential (−80 mV) for control condition (upper trace) and without *h*-channel (lower trace). (b) The impedance magnitude at the hyperpolarized resting membrane potential (−80 mV) for control condition (solid line) and without *h*-channel (dashed line). (c) At the depolarized resting membrane potential (−53 mV), the voltage response for control condition (upper trace) and without *h*-channel (lower trace). (d) The impedance magnitude at the depolarized resting membrane potential (−80 mV) for control condition (solid line) and without *h*-channel (dashed line). For removing Ca^2+^-channel, at hyperpolarized resting membrane potential (−80 mV), (e) the voltage response for control condition (upper trace) and without Ca^2+^-channel (lower trace) and (f) impedance magnitude for control condition (solid line) and without *h*-channel (circles). At depolarized resting membrane potential (−53 mV), (g) the voltage response for control condition (upper trace) and without Ca^2+^-channel (lower trace), and (h) impedance magnitude for control condition (solid line) and without Ca^2+^-channel (dashed line).

For the KCa-channel, despite the negativity of *L*_KCa1_ and *R*_KCa1_, the elements of other branches (Figure 4) take positive values (see Table 3) and the sum of currents of positive element branches is dominant in *I*_KCa_. This result denotes that negative feedback exists between the conductance of KCa-channel and membrane depolarization and implies that KCa-channel plays a resonating role in PD neurons. To visualize our argument and find the underlying mechanisms of ionic currents in the subthreshold resonant properties of PD neurons, the maximum conductance of KCa-channel is set to a large value (hundred times larger). The results are shown in Figure 10.

At the hyperpolarized resting membrane potentials, removing *h*-channel abolishes the resonant properties of voltage response (Figure 10(a)) and impedance profile (Figure 10(b)), whereas removing Ca^2+^-channels has no effect on voltage response (Figure 10(e)) and impedance profile (Figure 10(f)). These results agree with those of the HH-type model (Figures 8(a), 8(b), 9(a), and 9(b)). At depolarized resting membrane potentials, setting maximum conductance of *h*-channel to zero does not completely eliminate the resonance (Figures 10(c) and 10(d)), and removing Ca^2+^-channels (which eliminates KCa-channel) largely abolishes the resonance (Figures 10(g) and 10(h)).

These results agree with the perceptual behavior of resonance in these ranges of the membrane potentials. Therefore, *h*-channel and Ca^2+^-channels are dominant factors that influence hyperpolarized and depolarized membrane potentials, respectively. In the last part of this section, we examine the effect of flowing rate of ionic currents on resonance. Our aforementioned results show that *h*-channel, Ca^2+^-channels, and KCa-channel are crucial for the existence of membrane resonance. The inward current through *h*-channel, *I*_*h*_, is activated by hyperpolarizing in the membrane potential, which generates an inward excitatory current.

**Figure 11:**
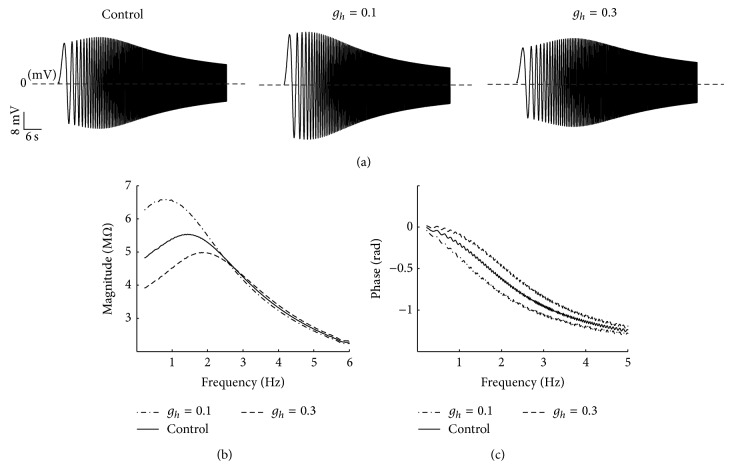
The effect of *h*-channel on the resonance in the equivalent RLC circuit model. (a) Voltage response for control condition (left trace), for *g*_*h*_ = 0.1 mS/cm^2^ (middle trace) and for *g*_*h*_ = 0.3 mS/cm^2^ (right trace). (b) Impedance magnitude for control condition (solid line), for *g*_*h*_ = 0.1 mS/cm^2^ (dashed line) and for *g*_*h*_ = 0.3 mS/cm^2^ (broken line). (c) Phase profile for control condition (solid line), for *g*_*h*_ = 0.1 mS/cm^2^ (dashed line) and for *g*_*h*_ = 0.3 mS/cm^2^ (broken line).

The effects of changing the maximum conductance of *h*-channel (*g*_*h*_) on voltage response (Figure 11(a)), IM (Figure 11(b)), and impedance phase (Figure 11(c)) are shown in Figure 11. Given its slow kinetics, *g*_*h*_ can follow the slow voltage change. Therefore, it is effective in damping the voltage change (Figure 11(a)). In Figure 11(b), decreasing *g*_*h*_ changes the impedance curve into the monotonically declining function and reduces the resonance frequency.

By contrast, increasing *g*_*h*_ transforms the resonance frequency into the high frequency range, changes the impedance curve into the unimodal curve, and decreases the maximum magnitude of impedance. This reduction may be attributed to the increase in resonant properties by *h*-channel (such as the effect of inductive component in circuit). Meanwhile, the amplifying property by Ca^2+^-channels is not changed. The phase also shows different frequency profiles during the alteration of *g*_*h*_. In Figure 11(c), the voltage always lags the current, except for a small range of frequencies under control condition, because of the maximum conductance value of the resonator current (*I*_*h*_).

**Figure 12:**
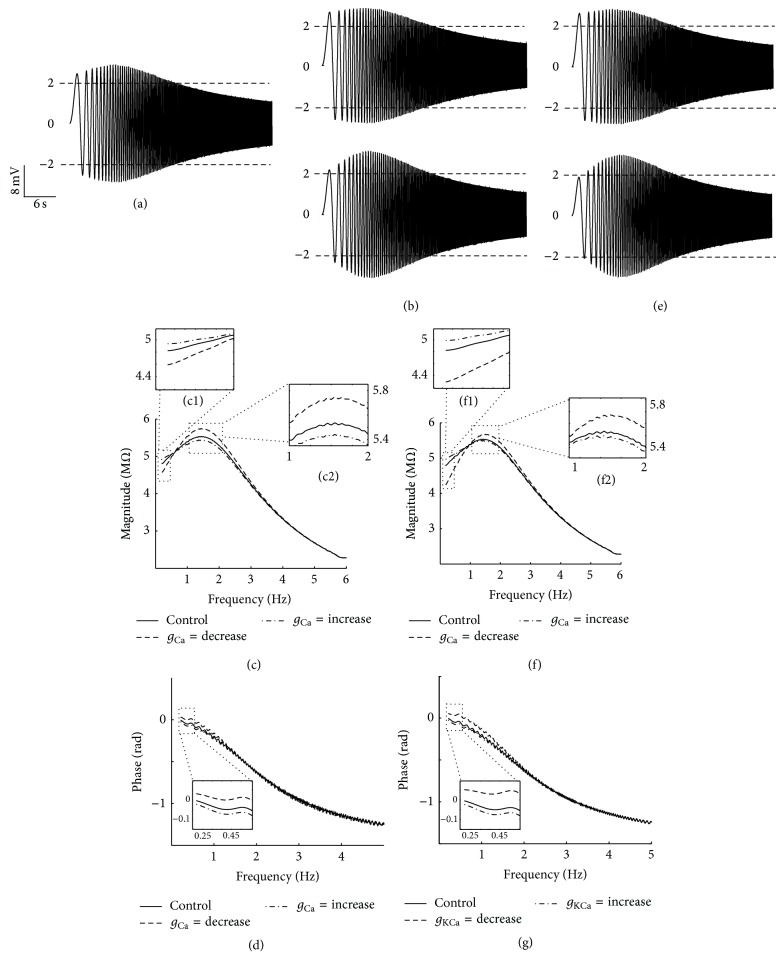
The effects of changing *g*_Ca_ and *g*_KCa_ in the equivalent RLC circuit model. (a) Voltage response for control condition. By decreasing *g*_Ca_ and *g*_KCa_, the resonance property of voltage response is abolished (both (b) and (e) upper trace of voltage responses). In contrast, by increasing *g*_Ca_ and *g*_KCa_, the resonance property of voltage response is magnified (both (b) and (e) lower trace of voltage responses). In impedance profile, by increasing *g*_Ca_ and *g*_KCa_, the impedance magnitude is increased (because of amplifying roles of *g*_Ca_) and the resonance frequency (*f*_res_) moved right because of the resonating role of *g*_KCa_ ((c) and (f)). The impedance phase shows positive phase area in control condition. The positive area increases by increasing *g*_Ca_ and *g*_KCa_ ((d) and (g)).

Figure 12 shows the effects of changing *g*_Ca_ and *g*_KCa_ on resonant property of equivalent RLC circuit model. The aforementioned results reveal that Ca^2+^-channels and KCa-channel act as the amplifier and resonator in PD neurons, respectively. However, the voltage responses are similarly changed by varying the maximum conductance of both channels (Figures 12(b) and 12(e)), such that reducing both maximum conductances decreases the IM and resonance frequency, as shown in Figures 12(c) and 12(f). By contrast, increasing both maximum conductances increases the resonance frequency and IM. To confirm our arguments about the roles of Ca^2+^-channels and KCa-channel in the resonance, we focus on two frequency ranges (0–0.5 Hz and 1-2 Hz) of IM (Figures 12(c) and 12(f)). In the frequency range of 0 to 0.5 Hz, changing *g*_KCa_ influences the impedance value more than changing *g*_Ca_ (compare Figures 12(c)(c1) and 12(f)(f1)). This result indicates that *g*_KCa_ has more tendencies to weaken or strengthen resonant properties than *g*_Ca_ (compare Figures 11(b) and 12(f); behavior of KCa-channel is roughly analogous to the one of *h*-channel in the frequency range from 0 to 0.5 Hz). Conversely, the value of IM changes more by varying *g*_Ca_ than *g*_KCa_ (compare Figures 12(c)(c2) and 12(f)(f2)) at 1-2 Hz. Accordingly, the value change may indicate the amplifying role of Ca^2+^-channels in PD neurons.

## 5. Conclusion

Previous* in vitro* studies have shown that the subthreshold oscillation of neural networks is closely related to the resonant properties of individual neurons. Moreover, subthreshold resonance oscillation is directly related to synaptic plasticity [8], which might be the foundation of learning and memory in the brain [11]. Thus, the evaluation of these properties in the HH-type model of neuron can be extremely helpful to understand the mechanisms underlying the coherent network oscillation and the fundamental mechanism of learning and memory in the brain. Among mathematical methods, the linearization method around equilibrium point is an effective approach to analyze the resonant properties of complex nonlinear systems such as neurons. Although the linearized model of neurons can be used only under the conditions that the membrane potential is fluctuating with a small amplitude [22], the main limitation of the linearized model (equivalent RLC electrical circuit model) is due to the fact that it is unable to mimic the neuronal behavior near the threshold for spike generation. That is, the equivalent RLC circuit model is effective only when the small-amplitude inputs are given to the model, because it is derived by truncating higher-order term more than second-order in Taylor expansion. Despite this limitation, a quantitative analysis of the linearized model of a neuron in the subthreshold region provides a temporal resolution that has not been investigated through* in vitro* or* in vivo* experiments. This point can be justified by two reasons. First, the effects of nonlinearities of membrane in the subthreshold region are not significant; second, the small amplitude input is always given to the neurons to evaluate their resonant properties [14]. Larger amplitude inputs lead to the appearance of other types of neural oscillations such as spiking and bursting [25]. In the present study, linearization method was used to investigate the effects of ionic currents on the resonant properties of PD neurons. In these neurons, the oscillatory properties of network activity around the preferred frequency can be obtained from the characteristics of single-neuron resonance behavior [12]. Thus, our results may serve as a basis to understand the properties of PD network oscillation. In this study, we linearized the HH-type dynamics and derived the equivalent RLC electrical circuit model of PD neurons. Then, Chirp current input was given to both the HH-type model and its equivalent RLC circuit model. The effects of individual ionic channel on the resonant properties of PD neurons were investigated by considering the voltage response and impedance profile of the equivalent RLC circuit.

### 5.1. Subthreshold Resonance Oscillation

Many neurons show subthreshold resonant properties to sinusoidal input current whose frequency changes in time. For example, the resonance frequency in cortical neurons appears between 3 and 12 Hz [26], in olive neurons between 3 and 10 Hz [4], and the 2–4 Hz range in thalamic neurons [27]. PD neurons show a resonant peak at lower than 2 Hz, thereby playing a vital role in determining the resonant properties in the pyloric network of crustaceans [12]. These properties are also affected by hyperpolarization-activated inward current (*I*_*h*_) and calcium currents (*I*_CaT_ and *I*_CaS_) together with *I*_KCa_. Our results showed that the resonance frequency is approximately 1.3 Hz in −55 mV (Figure 5), which agrees with the experimental results [12]. We also showed that the resonance behavior in the HH-type model depends on membrane potential, so that the strong hyperpolarizing resting potential causes the disappearance of resonance frequency, and the depolarizing resting potential increases their frequencies. Moreover, the maximum IM of resonance frequency appeared in −55 mV and reduced in both depolarizing and hyperpolarizing directions.

### 5.2. Roles of Ionic Channels

In general, the existence of resonance depends on the coexistence of low-pass and high-pass filter structures in the neurons. Low-pass filter is the passive membrane current and high pass-filter is the slow voltage-dependent current activated in the low frequency. In PD neurons, the voltage dependency of *h*-channel, Ca^2+^-channels (CaT- and CaS-channels), and KCa-channel and their activation/deactivation time constants determine the IM and resonance frequency range. Our simulated results for the equivalent RLC circuit model of PD neurons indicated that the *h*-channel plays a dominant role in determining the frequency preference range of the model in hyperpolarized potentials. By contrast, removing *h*-channel at depolarized potentials mainly abolished the low trace of voltage response and changed the resonant frequency, IM, and phase. This result agrees with experimental results [12]. From a physiological point of view, *h*-channel is slowly activated during the hyperpolarization of membrane potential inducing inward current that raises neuron depolarization toward its Nernst potential (−20 mV). Thus, this current opposes the potential change in the low frequency range and produces a negative feedback in combination with the passive membrane current. This phenomenon generates a preferred frequency band. By contrast, Ca^2+^-channels are activated by membrane depolarization, originating an inward Ca^2+^ current that depolarizes the membrane potential further and activates KCa-channel. This result indicates that activating calcium channels generates a positive feedback effect that amplifies voltage change. However, the resonance for the HH-type model was abolished by removing Ca^2+^-channels. From a physiological point of view, the calcium currents cannot be blocked without removing the calcium-dependent potassium current. Thus, the effects of Ca^2+^-channels on the resonant properties of PD neurons remain unclear. Using a modelling method, we showed that Ca^2+^-channels amplify the resonant properties of PD neurons and that *h*-channel and KCa-channel dominantly affect resonance. To visualize our argument, we increased the maximum conductance of KCa-channel (subsequently reducing the value of inductive components of KCa-channel in equivalent RLC circuit model) to transfer the resonance frequency to the high frequency range, which is generated by KCa-channel in the absence of *h*-channel (Figure 10). After this adjustment, our results showed that removing *h*-channel at hyperpolarized membrane potential abolishes resonance (Figures 10(a) and 10(b)), whereas removing Ca^2+^-channels and KCa-channel has no effect on resonant properties (Figures 10(e) and 10(f)). Conversely, removing *h*-channel at depolarized membrane potentials partially changes the resonant properties (Figures 10(c) and 10(d)), whereas removing Ca^2+^-channels and KCa-channel mainly abolishes the resonance (Figures 10(g) and 10(h)). The effect of the flowing rate of ionic currents on resonance was also investigated. Because of the slow kinetics of *h*-channel, it follows the slow potential change. Thus, it is effective in damping the potential change (Figure 11(a)). Moreover, decreasing *g*_*h*_ decreased the resonance frequency and changed the impedance curve into the monotonically declining function. By contrast, increasing *g*_*h*_ transferred impedance to the unimodal curve and increased resonance frequency (Figure 11).

The effects of changing the maximum conductances of Ca^2+^-channels and KCa-channel on the resonant properties of the equivalent RLC circuit model (Figure 12) were also examined. To confirm our arguments about the roles of equivalent RLC circuit model in resonance, we focused on two frequency ranges (0–0.5 Hz and 1-2 Hz) of IM (Figures 12(c) and 12(f)). In the frequency range of 0–0.5 Hz, changing *g*_KCa_ had more effect on impedance value than changing *g*_Ca_. This result indicates that *g*_KCa_ has more tendencies to weaken or strengthen resonant properties than *g*_Ca_. Conversely, the value of IM changed more by varying *g*_Ca_ than *g*_KCa_ in the frequency range of 1-2 Hz. This change may indicate the amplifying role of Ca^2+^-channels in PD neurons, which play the same role as NaP-channel in the entorhinal cortex layer II [28].

In conclusion, the effect of increasing Ca^2+^-channels on the resonance of PD neurons is mainly confined to the amplification, which accelerates the firing rates and promotes subthreshold oscillation in PD neurons. The inward calcium current (*I*_Ca_) also activates the KCa-channel that allows K^+^ efflux from intracellular to extracellular space and reduces the electrochemical gradient, which promotes membrane hyperpolarization. This result indicates that *I*_KCa_ significantly influences the resonant properties of PD neurons. Our result also showed that some of the resistance (*R*_*x*_ = 1/*g*_*x*_) and inductance values of the equivalent RLC circuit model were negative (see Section 4). The negativity of Ca^2+^-channels components mainly depends on the membrane potential along with the Nernst potential of the Ca^2+^-channels (*E*_Ca_). The negative components of KCa-channel components depend on *E*_Ca_ and both *τ*_*m*KCa_^∗^ and *τ*_Ca_. However, the sum of currents of positive branches was dominant in the behavior of KCa-channel. This result consequently confirmed the existence of negative feedback between conductance KCa-channel and membrane depolarization and implied that KCa-channel plays a resonating role in PD neurons. Based on these results, we suggest that the overall time constant of the membrane is directly affected by the calcium concentration in PD neurons. Further experimental investigations are required to understand this phenomenon.

## Figures and Tables

**Table 1:** Voltage and calcium dependency for the steady-state activation *m* and inactivation *h* of the currents.

	*m*, *h*	*x*_*∞*_	*τ*_*x*_, (msec)
*I*_*h*_	*m*	$\frac{1}{1+\exp\left[(V+48.5)/4.8\right]}$	$50+\frac{200}{1+\exp\left[-(V+42.2)/8.73\right]}$
*I*_CaT_	*m* ^3^	$\frac{1}{1+\exp\left[-(V+25)/7.2\right]}$	$1+\frac{9}{1+\exp\left[(V+58)/17\right]}$
	*h*	$\frac{1}{1+\exp\left[(V+36)/7\right]}$	$80+\frac{10}{1+\exp\left[(V+50)/17\right]}$

*I*_CaS_	*m* ^3^	$\frac{1}{1+\exp\left[-(V+22)/8.5\right]}$	$16-\frac{13.1}{1+\exp\left[-(V+25.1)/16.4\right]}$
*I*_KCa_	*m* ^4^	$\left[\frac{\left[\text{Ca}\right]}{\left[\text{Ca}\right]+30}\right]\frac{1}{1+\exp\left[-(V+51)/8\right]}$	$90.3-\frac{75.1}{1+\exp\left[-(V+46)/22.7\right]}$

**Table 2:** Parameter values for the model.

	*g*_*x*_ (*µ*S/cm^2^)	*E*_*x*_ (mV)
*I*_*h*_	0.219	−20
*I*_CaT_	2.25	—
*I*_CaS_	5.4	—
*I*_KCa_	150	−80
*I*_leak_	0.105	∗
*C*	12 (nF)	
[Ca]	*τ*_Ca_ = 300 (mS), Ca0 = 0.5 (*µ*M), *F* = 0.515 (*µ*M/nA)	

^*^The leak reversal potential is calculated by the steady-state values to remove the error, which occurs for zero-input case.

**Table 3:** The values of elements in RLC circuit model.

	*R*_*x*_ (MΩ)	*L*_*x*_ (H)
*I*_*h*_	*R*_*h*0_ = 0.066	
	*R*_*hm*_ = 0.01	*L*_*hm*_ = 0.0011

*I*_CaT_	*R*_CaT0_ = 133.2	
	*R*_*m*CaT_ = −2.98	*L*_*m*CaT_ = −0.01
	*R*_*h*CaT_ = 137.8	*L*_*h*CaT_ = 11.8

*I*_CaS_	*R*_CaS0_ = 22.5	
	*R*_*m*CaS_ = −0.6	*L*_*m*CaS_ = −0.007

*I*_KCa_	*R*_KCa0_ = 4543.3	
	*R*_KCa1_ = −83.3	*L*_KCa1_ = −5
	*R*_KCa2_ = 17.7	*L*_KCa2_ = 5.3
	*R*_KCa3_ = 2221	*L*_KCa3_ = 28.4
	*R*_KCa4_ = 4618.5	*L*_KCa4_ = 396
	*R*_KCa5_ = 83257	*L*_KCa5_ = 425

## Appendix

### A. Admittance of the Equivalent RLC Circuit

The admittance of equivalent RLC circuit is obtained in (14), (15), (16), and (21), which are the reverse of impedances elements related to CaT-, CaS-, *h*-, and KCa-channels, respectively. For voltage-dependent channels (CaT, CaS, and *h*), the resistance and inductance elements are obtained by

(A.1) $$\begin{array}{c} Z_{x}=L_{x}\cdot p+R_{x}, \end{array}$$

where *Z* is the impedance of each branch (Figure 2) and *p* is the Heaviside operator. Thus, the components of equivalent RLC circuit are obtained by the following.

#### A.1. Resistance and Inductance for* h*-Channel

Consider

(A.2) Rh0=1g¯h·mh∗,Rhm=1g¯h·V∗−Eh·dmh∞V/dV∗,Lhm=1g¯h·V∗−Eh/1/τmh∗·dmh∞V/dV∗.

#### A.2. Resistance and Inductance for CaT-Channel

Consider

(A.3) RCaT0=1g¯CaT·mCaT∗3·hCaT∗,RmCaT=g¯CaT·3·mCaT∗2·hCaT∗·V∗−ECadmCaT∞VdV∗WWWWW·dmCaT∞VdV∗−1,LmCaT=g¯CaT·3·mCaT∗2·hCaT∗·V∗−ECaτmCaT∗WWWWW·mCaT∗2τmCaT∗dmCaT∞VdV∗−1,RhCaT=g¯CaT·mCaT∗3·V∗−ECadhCaTVdV∗WWWWW·dhCaTVdV∗−1,LhCaT=g¯CaT·mCaT∗3·V∗−ECaτhCaT∗WWWWW·mCaT∗3τhCaT∗dhCaT∞VdV∗−1.

#### A.3. Resistance and Inductance for CaS-Channel

Consider

(A.4) RCaS0=1g¯CaS·mCaS∗3,RmCaS=3·g¯CaS·mCaS∗2·V∗−ECadmCaS∞VdV∗WWWWW·dmCaS∞VdV∗−1,LmCaS=3·g¯CaS·mCaS∗2·V∗−ECa1/τmCaS∗WWWWW·3.g¯CaS·mCaS∗21/τmCaS∗dmCaS∞VdV∗−1.

#### A.4. Resistance and Inductance for KCa-Channel

Consider

(A.5) RKCa01A,RKCa1=1Y1·τmKCa∗,LKCa1=1Y1,RKCa2=1Y2·τCa∗,LKCa2=1Y2,RKCa3=1Y3·τmCaS∗,LKCa3=1Y3,RKCa4=1Y4·τhCaT∗,LKCa4=1Y4,RKCa5=1Y5·τmCaT∗,LKCa5=1Y5,

where *Y*_*y*_ (*y* = 1,2, 3,4, 5) are given by

(A.6) Y1=B+C+D1/τCa−1/τmKCa∗+E1/τCa−1/τmKCa∗·1/τmCaS∗−1/τmKCa∗+G1/τCa−1/τmKCa∗·1/τhCaT∗−1/τmKCa∗+H1/τCa−1/τmKCa∗·1/τmCaT∗−1/τmKCa∗,Y2=C+D1/τmKCa∗−1/τCa+E1/τmKCa∗−1/τCa·1/τmCaS∗−1/τCa+G1/τmKCa∗−1/τCa·1/τhCaT∗−1/τCa+H1/τmKCa∗−1/τCa·1/τmCaT∗−1/τCa,Y3=E1/τmKCa∗−1/τmCaS∗·1/τCa−1/τmCaS∗,Y4=G1/τmKCa∗−1/τhCaT∗·1/τCa−1/τhCaT∗,Y5=H1/τmKCa∗−1/τmCaT∗·1/τCa−1/τmCaT∗,

and parameters *A*, *B*, *C*, *D*, *E*, *F*, and *G* are determined from (21) as follows:

(A.7) A=g¯KCa·mKCa∗4,B=4·g¯KCa·mKCa∗3·V∗−EK·dmKCa∞V,Ca/dV∗τmKCa∗,C=4·g¯KCa·mKCa∗3·V∗−EK·g¯CaS·mCaS∗3dmKCa∞V,CadCa∗WWWmCaS∗3·−F·dmKCa∞V,CadCa∗C=·τmKCa∗·τCa−1,D=4·g¯KCa·mKCa∗3·V∗−EK·g¯CaT·mCaT∗3·hCaT∗dmKCa∞V,CadCa∗WWW·−F·dmKCa∞V,CadCa∗D=·τmKCa∗·τCa−1,E=12·g¯KCa·mKCa∗3·V∗−EK·g¯CaS·mCaS∗2·−FdmCaSVdV∗WWW·V∗−ECa·dmKCa∞V,CadCa∗·dmCaSVdV∗E=·τmKCa∗·τCa·τmCaS∗−1,G=4·g¯KCa·mKCa∗3·V∗−EK·g¯CaT·mCaT∗3·−FdhCaTVdV∗WWW·V∗−ECa·dmKCa∞V,CadCa∗·dhCaTVdV∗G=·τmKCa∗·τCa·τhCaT∗−1,H=12·g¯KCa·mKCa∗3·V∗−EK·g¯CaT·mCaT∗2·hCaT∗·−FWWW·V∗−ECa·dmKCa∞V,CadCa∗·dmCaTVdV∗H=·τmKCa∗·τCa·τmCaT∗−1.
